# Early changes in the haemostatic and procoagulant systems after chemotherapy for breast cancer

**DOI:** 10.1038/sj.bjc.6604620

**Published:** 2008-09-02

**Authors:** C C Kirwan, G McDowell, C N McCollum, S Kumar, G J Byrne

**Affiliations:** 1 Department of Surgery, South Manchester University Hospitals Trust, Wythenshawe Hospital, Southmoor Road, Manchester M23 9LT, UK; 2 Department of Clinical Biochemistry, Manchester Royal Infirmary, Oxford Road, Manchester M13 9WL, UK; 3 Department of Pathology, Manchester Medical School, Oxford Road, Manchester M13 9PT, UK; 4 Department of Pathology, University of Manchester and Christie Hospital NHS Trust, Manchester Medical School, Oxford Road, Manchester M13 9PT, UK

**Keywords:** breast cancer, chemotherapy, venous thromboembolism, procoagulants

## Abstract

Venous thromboembolism (VTE) following breast cancer chemotherapy is common. Chemotherapy-induced alterations in markers of haemostasis occur during chemotherapy. It is unclear how rapidly this occurs, whether this is upregulated in patients developing VTE and whether changes predict for VTE. Markers of haemostasis, functional clotting assays and vascular endothelial growth factor were measured before chemotherapy and at 24 h, 4 days, 8 days and 3 months following commencement of chemotherapy in early and advanced breast cancer patients and in age- and sex-matched controls. Duplex ultrasound imaging was performed after 1 month or if symptomatic. Of 123 patients, 9.8% developed VTE within 3 months. Activated partial thromboplastin time (APTT), prothrombin time (PT), D-dimer, fibrinogen, platelet count, VEGF and fibrinogen were increased in cancer. Fibrinogen, D-dimer, VEGF and tissue factor were increased, at baseline, in patients subsequently developing VTE. D-dimer of less than 500 ng ml^−1^ has a negative predictive value of 97%. Activated partial thromboplastin time, PT and thrombin–antithrombin showed significantly different trends, as early as within 24 h, in response to chemotherapy in patients subsequently developing VTE. Markers of coagulation and procoagulants are increased, before chemotherapy, in patients who subsequently develop VTE. A group of patients at minimal risk of VTE can be identified, allowing targeted thrombopropylaxis to the higher risk group.

Venous thromboembolism (VTE) following breast cancer chemotherapy is not uncommon. In early breast cancer, VTE occurs in 5–10% of patients receiving chemotherapy (Weiss *et al*, 1981; Levine *et al*, 1988; von Tempelhoff *et al*, 1996), with a mortality of 0.2–0.5% (Weiss *et al*, 1981; Clahsen *et al*, 1994). Venous thromboembolism rises to approximately 18% in advanced breast cancer (Goodnough *et al*, 1984), with a mortality of 9%. Approximately two-thirds of all VTEs occur within 3 months of commencing chemotherapy (von Tempelhoff *et al*, 1996; Seward *et al* 1999); however, despite this, thromboprophylaxis is rarely used (Kirwan *et al*, 2003).

Previous work has demonstrated a hypercoagulable state in breast cancer patients, with elevated markers of coagulation, including thrombin–antithrombin (TAT) (Falanga *et al*, 1998; Ozyilkan *et al*, 1998), fibrinogen (Miller and Heilmann, 1988), D-dimer (Blackwell *et al*, 2000; Oberhoff *et al*, 2000) and tissue factor (TF) (Lwaleed *et al*, 1999; Ueno *et al*, 2000).

Several small studies have reported alterations in markers of coagulation in response to breast cancer chemotherapy, which support the development of a chemotherapy-induced hypercoagulable state (Canobbio *et al*, 1986; Rogers *et al*, 1988; Feffer *et al*, 1989; Rella *et al*, 1996; Pectasides *et al*, 1999). Several pathogenic mechanisms have been suggested such as increased expression or release of procoagulants and cytokines from damaged cells, a direct toxic effect on vascular endothelium or upregulation of platelet or monocyte activity. Although it is widely accepted that development of VTE is multifactorial, the increased rate of chemotherapy-induced VTE occurring in advanced breast cancer patients as compared with adjuvant patients suggests that other mechanisms may be occurring in the former group. It has been postulated that the direct toxic effect of chemotherapy on cancer cells may lead to increase in circulating tumour cell fragments or microparticles with associated procoagulant activity (Dvorak *et al*, 1983).

To date, the speed of onset of biochemical alterations in haemostasis remains to be elucidated. Moreover, no study has related cancer load to haemostatic and circulating procoagulant response to chemotherapy.

Preoperative D-dimer, prothrombin fragments 1 and 2 (PF1+2) and TAT have been shown to have some value in predicting postoperative VTE in patients undergoing major orthopaedic surgery (Bongard *et al*, 1994; Ginsberg *et al*, 1995; Cofrancesco *et al*, 1997; Lowe *et al*, 1999); however, markers of hypercoagulability and D-dimer were found to be of no use in predicting VTE in acutely ill medical patients (Crowther *et al*, 2005). Preoperative PF1+2, TAT and soluble fibrin do not predict postoperative DVT in colorectal cancer patients; however, postoperative (days 1–7) levels predict DVT, before a clinical diagnosis has been made (Iversen and Thorlacius-Ussing, 2002).

Although several studies have investigated haemostatic responses to chemotherapy at a biochemical level, no study to date has related these to the development of VTE, with a view to identifying patients at increased risk of thrombosis.

In this study, we prospectively followed advanced and early breast cancer patients commencing chemotherapy to establish early alterations in markers of haemostasis and procoagulants. This study was undertaken to investigate the effect of cancer burden on chemotherapy-induced changes in biomarkers of haemostasis and to assess the utility of chemotherapy-induced changes in biomarker concentration in predicting VTE.

## Materials and methods

### Patients

A total of 123 female patients (median age 52 (range 31–78) years) commencing chemotherapy for breast cancer were recruited. Of these, 87 were receiving adjuvant chemotherapy following curative surgery, and 36 were receiving chemotherapy for radiographically proven metastatic breast disease (Table 1). Patients were excluded if they were on anticoagulants, had a past history of VTE or had implanted vascular access devices.

### Control subjects

Sixty-eight age-matched female controls (median age 48 (range 31–78) years) with no history of cancer acted as control subjects.

### Protocol

A prospective cohort study was undertaken. Markers of haemostasis (TAT, fibrinogen, D-dimer, platelet count), functional clotting assays (prothrombin time (PT) and activated partial thromboplastin time (APTT)) and procoagulants (TF, cancer procoagulant (CP) and plasma vascular endothelial growth factor (pVEGF)) were measured before chemotherapy and at 24 h, 4 days, 8 days and 3 months following commencement of chemotherapy in all patients. A clinical assessment for VTE was performed at the same time points. Bilateral screening full-leg duplex ultrasound imaging was performed by accredited vascular scientists (Society of Vascular Technologists) in all patients 1 month following commencement of chemotherapy, and repeated if symptoms developed.

### Blood sampling and analytical methods

Atraumatic venous blood sampling was performed at the antecubital fossa, and all specimens were separated and stored within 2 h after being collected into tubes containing citrate and ETDA as anticoagulants. Citrate and EDTA samples were immediately taken onto ice, and serum samples were allowed to clot at room temperature. All samples (except full blood count and clotting screen) were centrifuged for 20 min at 4 °C and 2500 **g**, and the plasma or serum was removed from the cells. Serum and citrated plasma samples were then divided into 0.3 ml aliquots.

Platelet-depleted plasma (PDP) was also prepared for the analysis of VEGF as detailed: one of the citrated tubes was immediately plunged into ice and taken to the laboratory where the sample was centrifuged at 4 °C for 20 min at 3500 **g**. The supernatant was removed and recentrifuged for 20 min at 3500 **g** at 4 °C following which the PDP was aliquoted and the last 0.5 ml discarded. All samples were stored at −80 °C until analysis.

Prothrombin time (normal range 9–13.5 s), APTT (normal range 16.5–24.5 s) and fibrinogen (normal range 1.5–5.0 g l^−1^) were assayed by standard laboratory methods on ACL 3000 (Instrumentation Laboratory, Warrington, UK). Platelet count was measured using the Advia 120 Haematology System (Bayer Diagnostic, Newbury, UK). Serum TAT concentration was determined by a microplate immunoenzymatic method (Enzygnost® TAT micro ELISA, Dade Behring, Marburg, Germany), with a sensitivity of 1 *μ*g ml^−1^. Plasma D-dimer samples were analysed by a quantitative fully automated ELISA assay using the VIDAS® D-Dimer (bioMérieux, Marcy l’Etoile, France) system, with a sensitivity of 45 ng ml^−1^ and upper limit of normal of 500 ng ml^−1^. Plasma TF was analysed using an enzyme-linked immunosorbent assay (ELISA) (American Diagnostica Inc., Greenwich, CT, USA), with a sensitivity of 10 *μ*g ml^−1^. Cancer procoagulant was measured indirectly using a three-stage chromogenic assay to assess CP activity as described by Mielicki and co-workers (Mielicki *et al*, 1999). Platelet-depleted plasma VEGF was analysed using an ELISA by R&D Systems® (Oxon, UK), with a sensitivity of 9 *μ*g ml^−1^.

### Ethical approval

The study was approved by the South Manchester Local Research Ethics Committee and all patients gave written informed consent.

### Statistical methods

Data on PT, APTT platelet count and fibrinogen was parametric and thus reported as mean (confidence interval). Data on D-dimer, TAT, TF, CP and pVEGF was parametric after log conversion and so reported as geometric mean (confidence interval). Comparative group analysis (early, advanced breast cancer and controls) of prechemotherapy patient values was performed by ANOVA, with further analysis of subgroups using Scheffe. Comparative group analysis (VTE within 3 months, VTE free) of patient values was performed by independent *T*-test. Changes in patient serum or plasma values with chemotherapy as compared with pretreatment values were performed by paired *T*-test; however, to minimise errors induced by multiple tests, a repeated-measures analysis (Greenhouse Geiser correction) to compare trends over time in patients with and without VTE was used. Comparative group analysis (VTE within 3 months, VTE free) of changes in coagulation parameters with chemotherapy as compared with pretreatment values were performed by analysis of covariance. A significance of *P*<0.05 was used. Binary logistic regression to identify predictors of VTE was also performed. Analysis was performed on baseline data, and change from baseline. Appropriate corrections were made for cancer stage and age.

## Results

Of 123 breast cancer patients receiving chemotherapy, 12 (9.8%) patients developed VTE within 3 months of chemotherapy, of which 8 (66.7%) were symptomatic. Six of 36 (17%) metastatic breast cancer patients and 6 of 87 (6.9%) early breast cancer patients receiving adjuvant chemotherapy developed VTE. Development of VTE was not related to age, menopausal status or tumour receptor expression.

### Baseline data: before chemotherapy

Before chemotherapy, both APTT and PT were prolonged in advanced and early breast cancer patients, compared with controls (*P*=0.01 and 0.06 respectively, Table 2). D-dimer, fibrinogen, TF, platelet count and pVEGF were all increased in advanced breast cancer compared with controls. TAT showed a similar trend. D-dimer, fibrinogen and pVEGF were increased in advanced breast cancer compared with early breast cancer, with TAT showing a similar trend. D-dimer and fibrinogen were increased in early breast cancer compared with controls (Table 2).

### Baseline data: development of VTE before chemotherapy

Before chemotherapy, fibrinogen, D-dimer, TF and pVEGF were increased in patients who subsequently developed VTE within 3 months (fibrinogen: 4.9 (3.0–6.9) g l^−1^, 3.4 (3.2–3.7) g l^−1^, *P*=*0.002/0.1*, D-dimer: 1655 (834–3273) ng ml^−1^, 727 (631–836) ng ml^−1^, *P*=0.003; TF: 274 (115–654) *μ*g ml^−1^, 107 (86–134) *μ*g ml^−1^, *P*=0.03; pVEGF: 27.8 (14.3–54.1) *μ*g ml^−1^, 15.4 (13.5–17.7) *μ*g ml^−1^, *P*=*0.01*, VTE within 3 months and VTE free, respectively) (independent *T*-test). Similar trends were found when advanced breast cancer patients were analysed separately (fibrinogen: 4.1 (3.3–4.9) g l^−1^, 6.9 (3.7–10.1) g l^−1^, *P*=*0.01*; D-dimer: 2769 (1578–4859) ng ml^−1^, 1148 (806–1634) ng ml^−1^, *P*=0.03; TF: 271 (117–627) *μ*g ml^−1^, 164 (125–215) *μ*g ml^−1^, *P*=0.13; pVEGF: 19.5 (15.8–24.1) *μ*g ml^−1^, 49.6 (15.3–160.7) *μ*g ml^−1^, *P*=*0.004*, VTE within 3 months and VTE free, respectively).

In both early and advanced breast cancer patients, prechemotherapy fibrinogen and D-dimer are predictors for the development of VTE. A 1-g increase in fibrinogen doubles the risk of VTE (*P*=0.005), and every 1000 ng ml^−1^ increase in D-dimer is associated with a 1.8-fold increased risk of VTE (*P*=0.005).

Utilising the clinical cutoff for D-dimer of 500 ng ml^−1^, thrombosis could be predicted with a sensitivity of 92%, although specificity was low at 31%. However, most importantly, the negative predictive value was 97%. This has significant clinical relevance because nearly 30% of these breast cancer patients commencing chemotherapy had a D-dimer less than 500, and can therefore be almost guaranteed to be safe from VTE.

### Biomarker response to chemotherapy

The mean or geometric mean (CI) of coagulation parameters at baseline, 24 h, 4 days, 8 days and 3 months following chemotherapy is given in Table 3, and for patients subdivided into those developing VTE and remaining free of VTE, the values are given in Tables 4 and 5, respectively.

Analysing all patients together, irrespective of subsequent development of VTE, all molecules analysed showed a significant trend over time (repeated-measures analysis), except D-dimer and TF (repeated-measures analysis) (Table 3).

In patients with and without VTE, by 4 days following chemotherapy, platelet count was reduced; however, it remained within normal limits. Levels returned to baseline by 3 months (Table 3).

Activated partial thromboplastin time showed a marked shortening within 24 h of commencing chemotherapy; however, this was more pronounced in patients subsequently developing VTE (*P*=0.002). Although the shortening in APTT was maintained up to 3 months, the difference between those with and without VTE was not apparent after 24 h (Tables 4 and 5).

Conversely, PT demonstrated a prolongation in response to chemotherapy, which at days 4 and 8 was more marked in patients developing VTE (*P*=0.06 and 0.04, respectively) (Tables 4 and 5).

When patients who subsequently developed VTE were compared with patients who remained free of VTE, there was no difference in the response to chemotherapy of fibrinogen, D-dimer, CP and pVEGF (Tables 4 and 5).

Thrombin–antithrombin, in patients subsequently developing VTE, demonstrated a significant increase within 24 h in response to chemotherapy, which returned to baseline within 4 days (*P*=*0.02*) (Tables 4 and 5). Interestingly, at this time point, 4 patients had TAT values greater than 10-fold the upper confidence interval. Three out of these four patients subsequently developed VTE.

Tissue factor showed marked alterations in response to chemotherapy in those with and without VTE at 3 months. Tissue factor demonstrates a more marked decrease at 3 months, compared with baseline in patients subsequently developing VTE (*P*=0.02), even when corrections are made for cancer stage (Tables 4 and 5). Of these early alterations in coagulation in response to chemotherapy, only the prolongation of PT is an independent predictor for chemotherapy-induced thrombosis. The absence of an increase of PT at 8 days, relative to baseline, has a negative predictive power of 100%. As this occurs in 45% of patients, these individuals could be identified as no-risk for thrombosis on the basis of the change in PT at day 8.

## Discussion

This study supports previously published data on the frequency of VTE in breast cancer chemotherapy, with a rate of 17% in advanced breast cancer (Goodnough *et al*, 1984) and 8% (Weiss *et al*, 1981; Levine *et al*, 1988; von Tempelhoff *et al*, 1996) in early breast cancer patients receiving adjuvant therapy.

The increase in APTT and PT in breast cancer patients before chemotherapy suggests a paradoxical prolongation of clotting times, compared with non-cancer controls. Previous smaller studies have not demonstrated such a prolongation, but in all studies, the control groups were not matched to the study group. (Canobbio *et al*, 1986; Parmar *et al*, 1990; Mielicki *et al*, 1999; Oberhoff *et al*, 2000).

An increase in markers of the clotting system in advanced cancer, compared with early breast cancer, and early breast cancer compared with controls is demonstrated in this study by D-dimer and fibrinogen, confirming previous studies (Falanga *et al*, 1998; Blackwell *et al*, 2000; Oberhoff *et al*, 2000; Dirix *et al*, 2002). However, unlike previous research, in our study, the early breast cancer group has undergone complete tumour excision, but in spite of this, D-dimer levels remain elevated, perhaps implying a prolonged postoperative thrombotic response.

Elevated circulating TF levels in breast cancer have been described previously (Lwaleed *et al*, 1999; Ueno *et al*, 2000). Thrombocytosis has been described previously in cancer patients. (Sun *et al*, 1979; Pedersen and Milman, 1996).

The elevated prechemotherapy TAT levels in advanced breast cancer patients in this study support previous findings in breast (Falanga *et al*, 1998; Donati and Falanga, 2001), lung (Gabazza *et al*, 1992; Seitz *et al*, 1997) and colorectal cancer (Iversen *et al*, 1996; Iversen and Thorlacius-Ussing, 2002).

Consistent with previously published literature, pVEGF levels in this study are significantly elevated in advanced breast cancer patients (Adams *et al*, 2000). Interestingly, levels in the early breast cancer group (following apparent complete surgical resection) are comparable with controls. Previous literature report increased levels in early breast cancer patients before surgery, implying a resolution of elevated levels (Heer *et al*, 2001).

In this study, we have found, before chemotherapy, significantly elevated levels of D-dimer and fibrinogen in patients who subsequently develop VTE. Both markers are predictive for increased risk of VTE. Preoperative plasma levels of soluble fibrin polymers have been found to correlate with development of VTE following elective neurosurgery (Sonaglia *et al*, 1999).

A population-based prospective study has demonstrated a strong, positive relationship between D-dimer and development of future VTE (Cushman *et al*, 2003). Elevated presurgery D-dimer, before development of VTE, has been reported previously in patients undergoing hip surgery (Bongard *et al*, 1994; CoFrancesco *et al*, 1997). However D-dimer did not predict for VTE in medical or surgical patients admitted to ICU (Crowther *et al*, 2005), or in two studies of 71 and 60 ovarian cancer patients undergoing surgery (Olt *et al*, 1990; von Tempelhoff *et al*, 1997). In 50 early breast cancer patients undergoing chemotherapy, von Tempelhoff *et al* (1996) reports an elevated mean prechemotherapy D-dimer in the five patients subsequently developing VTE. In a small study investigating the anti-angiogenic compound SU5415 (an inhibitor of VEGF receptor 1 and 2), pretreatment TF levels were significantly elevated in the three patients who subsequently developed VTE, compared with the 17 patients without VTE (Kuenen *et al*, 2002), supporting our findings.

To date, no study has found a clinically useful test for predicting VTE; however, in this study, we demonstrate that a subgroup of patients (approximately one-third) can be considered as with minimal risk of VTE (D-dimer <500 ng ml^−1^ prechemotherapy), so that thromboprophylaxis can be targeted to a more precise high-risk group.

The almost universal, significant response of the measured molecules to breast cancer chemotherapy demonstrates that chemotherapy has a significant effect on coagulation and supports previous studies (Canobbio *et al*, 1986; Rogers *et al*, 1988; Feffer *et al*, 1989; Rella *et al*, 1996; Pectasides *et al*, 1999).

A shortening of APTT, in response to chemotherapy, has previously been reported; however, here we provide evidence that this response occurs within 24 h of commencing treatment and may be influential in VTE development. Both Pectasides and Canobbio (Canobbio *et al*, 1986; Pectasides *et al*, 1999) show shortening of functional clotting assay times equivalent to our 3-month findings. In lung cancer patients receiving chemotherapy, Gabazza *et al* (1992) reports an early shortening of APTT (at days 2, 5 and 7 following treatment) and a slightly later shortening of PT (at days 5, 7 and 14 following treatment). The fact worthy of note in the current study is that the shortening of APTT is more pronounced in the group subsequently developing VTE. Interestingly, Lowe *et al* (1999) report that a shortened preoperative APTT is the only independent predictor for post-hip surgery DVT in a study of 480 patients. The marked prolongation of PT at 8 days, occurring only in patients who subsequently develop VTE, has not been reported previously. Our current study is the first to identify such early alterations in functional clotting studies in response to breast cancer chemotherapy and, more importantly, that these alterations are more marked in patients subsequently developing VTE.

The finding that a lack of prolongation of PT from prechemotherapy to day 8 identifies a subgroup at no risk of VTE has profound clinical importance. A simple clotting study before commencement of treatment and at the 1-week outpatient appointment may half the number of patients that require thromboprophylaxis.

It is surprising that products of intravascular coagulation, such as fibrinogen and D-dimer show no significant alteration in response to chemotherapy in the prothrombotic VTE group.

The early changes we have demonstrated in clotting, particularly those changes occurring within 24 h (TAT and APTT), are too rapid to be caused by immobility secondary to chemotherapy-induced malaise, or biochemical and fluid alterations with emesis. The peak onset of VTE following total hip replacement is 4 days (Sikorski *et al*, 1981). The true time of onset for chemotherapy-induced VTE would require more extensive screening than was performed in this study; however, early haemostatic responses to chemotherapy may further upregulate a haemostatic system that is already induced, due to cancer and recent surgery. A small study of 16 advanced cancer patients treated with chemotherapy demonstrated an increase in plasma fibrinopeptide A (cleaved from fibrinogen by thrombin) within 45 min of chemotherapy administration; however, this response was abolished in 8 patients given a second course of chemotherapy when treatment was preceeded by heparin infusion (Edwards *et al*, 1990).

In conclusion, our large prospective study was the first study to look at early alterations in haemostasis following breast cancer chemotherapy, and the first to screen for VTE and relate such alterations to development of VTE. We have confirmed that chemotherapy-induced alterations occur early, within 24 h, of chemotherapy. Early alterations in functional clotting assays are more marked in patients subsequently developing VTE. Early use of thromboprophylaxis, perhaps even a single dose administered before chemotherapy, may abolish this rapid haemostatic response. We also present a method of identifying a subgroup of patients at minimal risk of VTE, thus allowing targeted thromboprophylaxis. A trial of single-dose thromboprophylaxis in this subset may be warranted.

## Figures and Tables

**Table 1:** Chemotherapy regimens used in breast cancer patients

**Chemotherapy regimen**	**Number of patients**
*Adjuvant regimens*	
5-Fluorouracil, epirubicin, cyclophosphamide	65
Cyclophosphamide, methotrexate, 5-fluorouracil	15
Epirubicin, cyclophosphamide	4
Epirubicin	3
	
*Metastatic regimens*	
Docetaxol	15
Cyclophosphamide, methotrexate, 5-fluorouracil	8
Epirubicin, docetaxol	6
Vinorelbine, mitomycin	3
Epirubicin	2
5-Fluorouracil, epirubicin, cyclophosphamide	1
Vinorelbine, 5-fluorouracil	1

**Table 2:** Baseline biomarker results before chemotherapy

**Coagulation marker**	**Advanced breast cancer**	**Early breast cancer**	**Control**	***P* ANOVA (Scheffe-showing paired comparisons)**
APTT secs, mean (CI) ***(n)***	22.6 (21.4–23.7)^*^ ***(26)***	23.2 (22.6–23.7)^†^ ***(77)***	20.7 (20.2–21.3)^*^^†^ ***(38)***	<0.001 (* 0.01, ^†^<0.001)
PT secs, mean (CI) ***(n)***	11.7 (11.4–12.1) ***(26)***	11.7 (11.5–11.8) ***(77)***	11.4 (11.2–11.6) ***(38)***	*0.06*
TAT *μ*g/ml, geometric mean (CI) ***(n)***	9.2*^†^ (4.7–18.1) ***(14)***	4.2* (2.8–6.4) ***(11)***	4.2^†^ (2.6–6.7) ***(13)***	0.05 (* 0.1, ^†^0.1)
Fibrinogen g/l, mean (CI) ***(n)***	4.5 (3.7–5.3)^*^^†^ ***(21)***	3.3 (3.1–3.5)^*^^‡^ ***(73)***	2.7 (2.5–3.0)^†^^‡^ ***(38)***	<0.001 (*<0.001, ^†^*<0.001,* ^‡^*0.05)*
D-dimer ng/ml, geometric mean (CI) ***(n)***	1334.9 (969.5–1837.8)^*^^†^ ***(35)***	668.7 (584.8–764.5)^*^^‡^ ***(87)***	287.9 (248.7–333.3)^†‡^ ***(61)***	<0.001 (*<0.001, ^†^*<0.001,* ^‡^<0.001 )
Platelet count × 10^9^/l, mean (CI) ***(n)***	326.7^*^ (286.9–366.5) ***(36)***	309.6 (293.0–326.2) ***(87)***	278.6 (257.7–299.6)^*^ ***(45)***	0.04 (*0.05)
TF *μ*g/ml, geometric mean (CI) ***(n)***	179.2 (139.9–229.6)^*^ ***(36)***	92.1 (67.8–125.1) ***(85)***	52.5 (30.8–89.6)^*^ ***(61)***	0.001 (*0.001)
CP mU, geometric mean (CI) ***(n)***	28.3 (24.8–32.3)* ***(33)***	33.1 (30.7–35.6)* ***(78)***	28.8 (25.3–32.8) ***(27)***	0.04 (*0.09)
pVEGF *μ*g/ml, geometric mean (CI) ***(n)***	22.8 (17.6–29.4)*^†^ ***(35)***	14.2 (12.1–16.6)* ***(86)***	15.1 (12.7–17.9)^†^ ***(61)***	0.004 (*0.01, ^†^*0.03)*

Analysis of the difference between groups used analysis of variance (ANOVA). Where differences were found, further analysis (between pairs of groups, with respective pairs for each molecule marked with ^*^,^†^, ^‡^ and ^§^) was performed using Scheffe (95% confidence interval (CI)).

**Table 3:** Alterations in biomarker parameters induced by chemotherapy in breast cancer patients

**Procoagulant/ adhesion molecule**	**Prechemotherapy *(n)***	**Day 1 (post-chemotherapy) *(n)***	**Day 4 (post-chemotherapy) *(n)***	**Day 8 (post-chemotherapy) *(n)***	**3 months (post-chemotherapy) *(n)***	**Trend over time (repeated measures, GG)**
APTT s, mean (CI)	23.0 (22.5–23.5) ***(103)***	21.6 (21.1–22.0) ***(108)***	21.4 (21.0–21.8) ***(107)***	21.8 (21.3–22.3) ***(108)***	21.0 (20.5–21.5) ***(100)***	*P<0.001*
PT s, mean (CI)	11.7 (11.5–11.8) ***(103)***	11.9 (11.7–12.0) ***(108)***	11.7 (11.6–11.9) ***(107)***	11.9 (11.7–12.0) ***(108)***	11.5 (11.3–11.7) ***(98)***	*P<0.001*
TAT *μ*g ml^−1^, geometric mean (CI)	6.6 (4.3–10.0) ***(25)***	11.3 (5.9–21.4) ***(24)***	6.6 (4.5–9.6) ***(24)***	5.6 (4.0–7.8) ***(24)***	4.9 (3.1–7.7) ***(20)***	*P*=*0.04*
Fibrinogen g l^−1^, mean (CI)	3.6 (3.3–3.8) ***(94)***	3.3 (3.0–3.5) ***(98)***	3.2 (2.9–3.4) ***(99)***	3.4 (3.2–3.7) ***(104)***	4.1 (3.8–4.3) ***(91)***	*P<0.001*
D-dimer ng ml^−1^, geometric mean (CI)	815.3 (707.8–939.3) ***(122)***	845.1 (729.3–979.1) ***(115)***	786.7 (673.7–918.7) ***(115)***	788.8 (680.5–914.3) ***(116)***	763.3 (650.9–895.2) ***(109)***	*P*=*0.4*
Platelet count × 10^9^ l^−1^ mean (CI)	314.6 (298.4–330.9) ***(123)***	306.1 (287.2–325.0) ***(99)***	263.3 (249.7–276.8) ***(108)***	242.3 (227.3–257.2) ***(117)***	310.0 (285.0–334.0) ***(112)***	*P<0.001*
TF *μ*g ml^−1^, geometric mean (CI)	112.3 (89.0–141.6) ***(121)***	116.3 (91.2–148.2) ***(116)***	97.8 (76.9–124.3) ***(113)***	97.3 (77.3–122.4) ***(115)***	94.1 (74.0–119.8) ***(107)***	*P*=*0.3*
CP mU, geometric mean (CI)	31.6 (29.6–33.7) ***(111)***	36.4 (34.1–38.8) ***(109)***	34.4 (32.2–36.7) ***(104)***	32.0 (29.8–34.3) ***(108)***	30.9 (28.8–33.1) ***(100)***	*P<0.001*
pVEGF *μ*g ml^−1^, geometric mean (CI)	16.4 (14.2–18.8) ***(120)***	14.9 (13.2–16.9) ***(115)***	16.3 (14.0–18.0) ***(112)***	20.5 (18.3–13.0) ***(114)***	21.6 (18.8–24.9) ***(106)***	*P<0.001*

**Table 4:** Alterations in biomarker parameters induced by chemotherapy in breast cancer patients developing VTE within 3 months of chemotherapy

**Coagulation marker**	**Prechemotherapy *(n)***	**Day 1 (post-chemotherapy) *(n)***	**Day 4 (post-chemotherapy) *(n)***	**Day 8 (post-chemotherapy) *(n)***	**3 months (post-chemotherapy) *(n)***
APTT secs, mean (CI)	22.2 (18.7–25.6) ***(9)***	19.8 (18.2–21.3) ***(9)***	21.1 (19.4–22.7) ***(11)***	21.6 (19.4–23.7) ***(8)***	21.0 (18.6–23.4) ***(9)***
PT secs, mean (CI)	11.5 (11.0–12.0) ***(9)***	11.9 (11.5–12.4) ***(11)***	11.9 (11.4–12.4) ***(11)***	12.1 (11.3–12.9) ***(9)***	11.8 (10.9–12.6) ***(7)***
TAT *μ*g ml^−1^, geometric mean (CI)	8.9 (2.4–32.5) ***(8)***	28.6 (4.9–167.1) ***(8)***	6.3 (2.4–16.4) ***(8)***	5.2 (2.4–11.1) ***(8)***	3.9 (2.5–5.9) ***(7)***
Fibrinogen g l^−1^ mean(CI)	4.9 (3.0–6.9) ***(7)***	4.2 (2.5–5.9) ***(8)***	4.2 (2.9–5.5) ***(10)***	4.4 (3.0–5.7) ***(7)***	5.6 (4.5–6.8) ***(8)***
D-dimer ng ml^−1^, geometric mean (CI)	1618.6 (979.0–2676.1) ***(12)***	1653.1 (996.0–2743.8) ***(11)***	1297.1 (585.2–2875.1) ***(11)***	1301.9 (642.4–2638.6) ***(11)***	966.7 (504.9–1850.8) ***(9)***
Platelet count × 10^9^ l^−1^ mean (CI)	342.5 (265.8–419.2) ***(12)***	338.9 (246.4–431.4) ***(10)***	269.6 (225.5–313.8) ***(11)***	274.0 (216.8–331.2) ***(11)***	386.2 (238.3–534.1) ***(10)***
TF *μ*g ml^−1^, geometric mean (CI)	231.1 (104.1–513.2) ***(11)***	228.7 (107.1–488.5) ***(10)***	190.9 (96.9–376.4) ***(10)***	192.9 (82.2–452.5) ***(10)***	81.4 (22.9–289.5) ***(8)***
CP mU, geometric mean (CI)	31.4 (26.6–37.1) ***(11)***	35.7 (28.4–44.9) ***(11)***	32.5 (26.3–40.1) ***(11)***	28.2 (22.9–34.7) ***(11)***	27.8 (21.2–36.5) ***(9)***
pVEGF *μ*g ml^−1^, geometric mean (CI)	27.8 (14.3–54.1) ***(12)***	19.5 (10.4–36.6) ***(11)***	19.0 (9.4–38.3) ***(11)***	23.4 (13.2–41.5) ***(11)***	18.7 (13.7–25.5) ***(9)***

**Table 5:** Alterations in biomarkers induced by chemotherapy in breast cancer patients remaining free of VTE within 3 months of chemotherapy

**Coagulation marker**	**Prechemotherapy *(n)***	**Day 1 (post-chemotherapy) *(n)***	**Day 4 (post-chemotherapy) *(n)***	**Day 8 (post-chemotherapy) *(n)***	**3 months (post-chemotherapy) *(n)***
APTT s, mean (CI)	23.1 (22.6–23.6) ***(94)***	21.8 (21.3–22.2) ***(97)***	21.5 (21.1–21.9) ***(96)***	21.8 (21.3–22.3) ***(99)***	21.0 (20.5–21.5) ***(91)***
PT s, mean (CI)	11.7 (11.6–11.9) ***(94)***	11.9 (11.7–12.0) ***(97)***	11.7 (11.6–11.9) ***(96)***	11.8 (11.7–12.0) ***(99)***	11.5 (11.3–11.7) ***(91)***
TAT *μ*g ml^−1^, geometric mean (CI)	5.7 (4.0–8.0) ***(17)***	7.1 (4.4–11.3) ***(16)***	6.7 (4.5–10.2) ***(16)***	5.8 (3.8–8.6) ***(16)***	5.6 (2.8–11.2) ***(13)***
Fibrinogen g l^−1^, mean(CI)	3.4 (3.2–3.7) ***(87)***	3.2 (2.9–3.4) ***(90)***	3.1 (2.8–3.3) ***(89)***	3.4 (3.1–3.6) ***(97)***	3.9 (3.6–4.2) ***(83)***
D-dimer ng ml^−1^, geometric mean (CI)	756.6 (655.9–872.7) ***(110)***	787.1 (677.5–914.5) ***(104)***	746.3 (640.7–869.1) ***(104)***	748.4 (646.1–867.0) ***(105)***	747.2 (632.5–882.7) ***(100)***
Platelet count × 10^9^ l^−1^ mean (CI)	311.6 (295.2–328.0) ***(111)***	302.4 (283.4–321.4) ***(89)***	262.5 (248.1–277.0) ***(97)***	239.0 (223.4–254.5) ***(106)***	302.5 (279.6–325.5) ***(102)***
TF *μ*g ml^−1^, geometric mean (CI)	104.4 (82.0–133.1) ***(110)***	109.1 (84.5–140.9) ***(106)***	91.7 (71.1–118.2) ***(103)***	91.2 (71.8–115.7) ***(105)***	95.3 (74.3–122.1) ***(99)***
CP mU, geometric mean (CI)	31.6 (29.4–33.9) ***(100)***	36.5 (34.1–39.1) ***(98)***	34.6 (32.3–37.1) ***(93)***	32.4 (30.1–34.9) ***(97)***	31.2 (29.0–33.6) ***(91)***
pVEGF *μ*g ml^−1^, geometric mean (CI)	15.4 (13.5–17.7) ***(108)***	14.5 (12.9–16.4) ***(104)***	16.0 (13.7–18.7) ***(101)***	20.2 (18.0–22.7) ***(103)***	21.9 (18.8–25.5) ***(97)***
